# Metal-Assembled Collagen Peptide Microflorettes as Magnetic Resonance Imaging Agents

**DOI:** 10.3390/molecules28072953

**Published:** 2023-03-26

**Authors:** Dawn Ernenwein, Iris Geisler, Anna Pavlishchuk, Jean Chmielewski

**Affiliations:** Department of Chemistry, Purdue University, West Lafayette, IN 47907, USA

**Keywords:** peptide, supramolecular assembly, collagen, microflorettes, MRI contrast agents, gadolinium, DOTA

## Abstract

Magnetic resonance imaging (MRI) is a medical imaging technique that provides detailed information on tissues and organs. However, the low sensitivity of the technique requires the use of contrast agents, usually ones that are based on the chelates of gadolinium ions. In an effort to improve MRI signal intensity, we developed two strategies whereby the ligand DOTA and Gd(III) ions are contained within Zn(II)-promoted collagen peptide (**NCoH**) supramolecular assemblies. The DOTA moiety was included in the assembly either via a collagen peptide sidechain (**NHdota**) or through metal–ligand interactions with a His-tagged DOTA conjugate (**DOTA-His_6_**). SEM verified that the morphology of the **NCoH** assembly was maintained in the presence of the DOTA-containing peptides (microflorettes), and EDX and ICP-MS confirmed that Gd(III) ions were incorporated within the microflorettes. The Gd(III)-loaded DOTA florettes demonstrated higher intensities for the T1-weighted MRI signal and higher longitudinal relaxivity (r_1_) values, as compared to the clinically used contrast agent Magnevist. Additionally, no appreciable cellular toxicity was observed with the collagen microflorettes loaded with Gd(III). Overall, two peptide-based materials were generated that have potential as MRI contrast agents.

## 1. Introduction

Magnetic resonance imaging (MRI) is a non-invasive diagnostic tool that allows for 3D imaging with high spatial and temporal resolution. The technique is suitable for the detection of various disease states and tissue abnormalities located within in the body without the need for ionizing radiation [1,2,3]. Magnetic resonance images are obtained by aligning the protons of water molecules within the body with an applied magnetic field and monitoring the time and energy release associated with the realignment of protons after a radio frequency pulse [4,5]. Additionally, the use of paramagnetic contrast agents influences the relaxation rates of protons in nearby water molecules. These agents significantly enhance contrast, leading to better quality images, and may provide valuable information when directed to specific in vivo targets [6,7,8].

Efficient paramagnetic contrast agents decrease the longitudinal relaxation time, T1, resulting in bright T1-weighted MRI images. Since the effectiveness of such contrast agents depends on the number of unpaired electrons, compounds containing metal ions with half-filled electron shells and quenched orbital contributions to the magnetic moment are the most promising candidates for T1 imagining purposes. For example, Gd(III) (*f^7^*) and high-spin Mn(II) and Fe(III) (*d^5^*) ions are of particular interest as potential contrast agents for MRI [9,10,11]. In addition to a high-spin ground state, good contrast agents should demonstrate long electron spin relaxation times, high stability, good solubility in water, low toxicity and fast excretion [12,13]. Gd(III) complexes with strongly chelating polycarboxylate ligands, possessing coordination sphere water molecules able to exchange with water molecules in tissues, currently dominate the field of contrast agents for MRI [14,15]. Until recently, Gd(III) contrast agents were considered to be safe; however, recent studies have demonstrated that Gd(III) compounds can remain in the body for a time and, in some cases, cause the development of nephrogenic systemic fibrosis [16,17,18]. In order to address the health risks connected with these Gd(III)-containing agents, research has focused on the creation of novel contrast agents with improved sensitivity that may potentially decrease the needed dose for patients.

Higher imaging sensitivity can be obtained by reaching a higher T1 relaxivity, which depends upon the hydration number of the contrast agent, rotational correlation time and water residency time [19]. A substantial decrease in rotation can be achieved by using molecules with a higher molecular weight and size. Recent studies have demonstrated that linking contrast agents to large molecules and assemblies (i.e., dextran [20], dendrimers [21,22,23], liposomes [24,25,26], nanoparticles [27,28], fullerenes [29], micelles [30,31], peptides [32,33,34] and proteins [35,36,37]) can substantially increase T1 relaxivity. In addition, the integration of Gd(III) ions within various macromolecules can lead to the formation of multiple paramagnetic centers, thereby conferring enhanced sensitivity [38,39,40,41]. In this regard, large assemblies with a spherical morphology rotate more isotropically and provide higher relaxivity values, as compared to linear assemblies that have faster rotation around their long axis [19,42].

The creation of highly sensitive peptide-based materials with a spherical morphology is of particular interest for MRI purposes, since many peptide-linked contrast agents have demonstrated selective binding to biotargets, including fibrin and collagen [42,43,44,45]. Peptide-based materials, such as peptide hydrogels, nanofibers and nanofibrils, have been investigated as potential biocompatible bioimaging agents [46]. Supramolecular peptide hydrogels associated with Gd(III) complexes have demonstrated a substantial increase in longitudinal relaxivity (r_1_); however, the potential release of Gd(III) ions from such assemblies limits their future application [47]. Recent studies with peptide-based nanofibers have revealed that such assemblies provide bright MRI images and, in some cases, demonstrate efficient accumulation in tumors [48,49]. Peptide amphiphiles that self-assemble into nanofibers [50], nanofibrils [51], liposomes [52] and micelles [53] demonstrate improvement in relaxivity values, as compared to commercial contrast agents, the latter being used in the design of targeted contrast agents [53]. A short DOTA-containing peptide that self-assembles into nanoparticles in the presence of furin in tumor cells led to an increase in relaxivity upon aggregation [54]. Inorganic paramagnetic nanoparticles decorated with peptides usually possess spherical symmetry and may provide sensitive imaging and targeted delivery of contrast agents [55]. However, the number of assemblies that have a spherical morphology formed solely with peptides remains limited. Collagen-based assemblies have been well studied due to the high abundance of the protein, biocompatibility, and the number of potential applications, including regenerative medicine and drug delivery [56]. With these systems in mind, we turned to a metal-triggered collagen peptide (**NCoH**) that forms microflorette particles [57,58,59]. Previous studies have shown that the combination of **NCoH** with metal ions can result in the formation of spherical microflorettes, a morphology that is of particular interest for the creation of MRI contrast agents [57]. Therefore, herein, we disclose two designs for the incorporation of Gd(III) into these microflorettes and detail the resulting MRI activity.

## 2. Results and Discussion

### 2.1. Design of MRI Active Collagen Peptide Microflorettes

**Figure 1:**
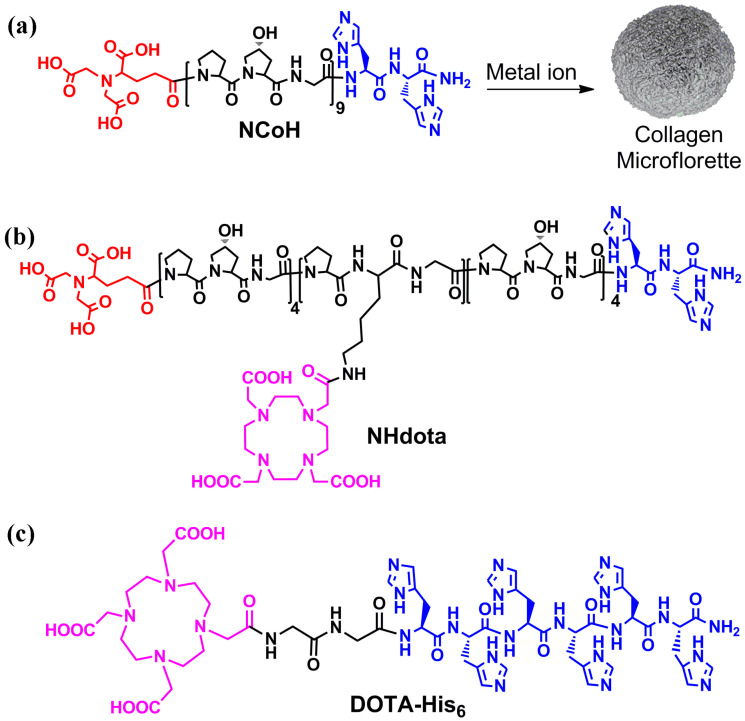
Structures of metal-triggered self-assembling peptides (**a**) **NCoH**, (**b**) **NHdota** and (**c**) **DOTA-His_6_**, with the nitrilotriacetic acid in red, histidines in blue and DOTA in pink. (**a**) Metal-promoted assembly of **NCoH** into microflorettes is shown.

Collagen-mimetic peptides based on repeating Pro-Hyp-Gly (POG) sequences have been extensively studied, as these sequences form a left-handed polyproline type II helix that assembles into right-handed triple helices with distinct structural stability [60]. The incorporation of ligands for metal ions within the collagen peptide triple helices has been shown to promote the higher order assembly of this building block into numerous morphologies, including fibers, disks, hollow cages and microflorettes [61]. With this in mind, the design of peptide assemblies with MRI activity started with a metal-triggered, self-assembling collagen peptide, **NCoH** (Figure 1a) [57]. This peptide contains a (POG)_9_ core with the N- and C-termini functionalized with nitrilotriacetic acid (NTA) and di-histidine ligands, respectively. Upon the addition of a range of metal ions to **NCoH**, spherical, ruffled collagen microflorettes were generated (Figure 1a).

**Figure 2:**
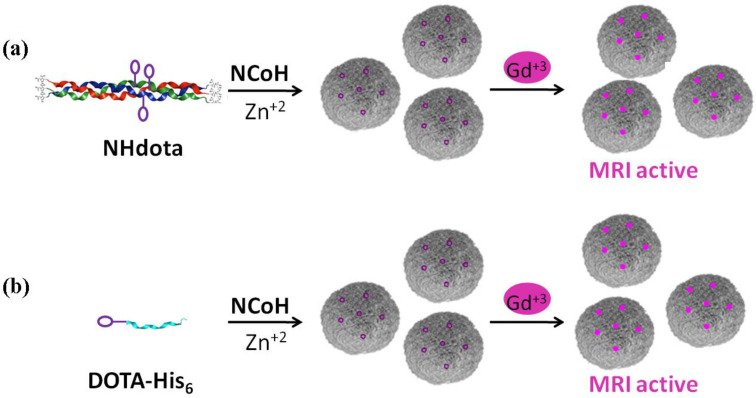
Strategies used for the generation of Gd(III)-loaded microflorettes with (**a**) **NHdota** peptides and (**b**) **DOTA-His_6_** peptides.

To prepare MRI active microflorettes, a Gd(III) chelating ligand was installed within the **NCoH** peptide sequence. Macrocyclic structures are tighter binders of Gd(III); therefore, the polydentate macrocycle 1,4,7,10-tetraazacyclodecane-1,4,7-10-tetraacetic acid (DOTA) was incorporated within the Zn(II)-triggered peptide microflorettes using two different strategies (Figure 2). In our first strategy, the DOTA ligand was incorporated at a central lysine residue within the middle of the (POG)_9_ collagen sequence of **NCoH**, yielding **NHdota** (Figure 1b), which could be used in conjunction with **NCoH** to form zinc-dependent assemblies (Figure 2a). In our second approach, we took advantage of available metal–ligand interactions as the florettes formed, in order to incorporate a His-tagged DOTA ligand, **DOTA-His_6_** (Figure 1c). This would introduce DOTA within the particles in a Zn(II)-triggered manner (Figure 2b), as we have previously demonstrated with His-tagged fluorescent proteins [58]. These two strategies have the potential to produce DOTA-containing microflorettes that could then be charged with Gd(III), so as to yield spherical particles with a high peptide content and an abundance of DOTA-Gd(III) moieties. The extensive hydrogen bonds within the amide backbone of the collagen peptides of the microflorettes may allow for an increased dynamic exchange of bulk water with inner sphere water molecules on Gd(III), thereby resulting in more active MRI agents. Recently, it was demonstrated that collagen peptide assemblies with lanthanide(III) ions result in the formation of luminescent nanofibers that may have potential for other bioimaging techniques [62,63,64]. In addition, it has been shown that collagen-based spherical assemblies with micron particle sizes possess the ability to serve as delivery agents [65,66].

### 2.2. Synthesis and Higher Order Assembly of Collagen Mimetic Peptides

The peptides, **NHdota**, **DOTA-His_6_** and **NCoH**, were synthesized on the H-Rink Amide ChemMatrix solid support using Fmoc-based protection and HBTU coupling chemistry. DOTA was incorporated into **NHdota** via a Lys residue in the central peptide triad. The peptides were cleaved from the resin with a TFA cocktail, purified to homogeneity by reverse-phase HPLC and characterized by MALDI-TOF mass spectrometry (MALDI MS expected/observed: **NCoH**: 2941/2937; **NHdota**: 3341.5/3336.0; **DOTA-His_6_**: 1339.6/1341.0). Circular dichroism (CD) was used to verify that the central addition of the DOTA moiety did not preclude **NHdota** from forming the expected collagen triple helix, and to determine its thermal stability. The CD spectrum of **NHdota** displayed a maximum molar ellipticity at 225 nm that is indicative of the PPII structure of collagen-like peptides (data not shown). Cooperative triple helix unfolding was observed for **NHdota** with a melting temperature of about 55 °C, a value that is slightly higher than the parent **NCoH** (~50 °C) [57].

**Figure 3:**
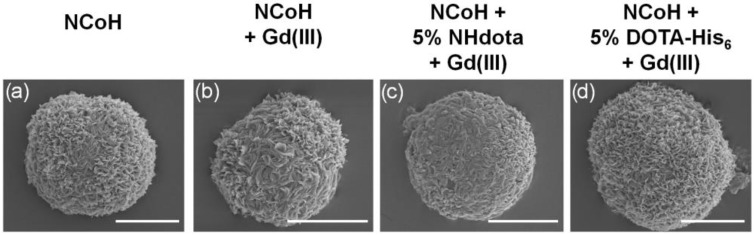
Scanning electron microscopy images of microflorettes generated with (**a**) 1 mM of **NCoH** and 400 µM of Zn(II), (**b**–**d**) followed by the addition of Gd(III) to pre-formed microflorettes composed of (**b**) **NCoH**, (**c**) 5% **NHdota** and (**d**) 5% **DOTA-His_6_** (scale bars = 10 µm).

Microflorettes form upon combining **NCoH** and Zn(II) (1.0 and 0.4 mM, respectively) in MOPS buffer (pH 7.1) (Figure 3a) [57]. Interestingly, the addition of Gd(III) to **NCoH** produced no higher order assembly under identical conditions. Therefore, to include Gd(III) into microflorettes, we employed the two strategies shown in Figure 2. The DOTA-containing peptides, **NHdota** or **DOTA-His_6_** (5% each), were added to **NCoH,** followed by the addition of Zn(II). After 24 h, the assembly was collected and washed by centrifugation, and scanning electron microscopy (SEM) was performed to verify microflorette formation. In both cases, the ruffled surface morphology of the microflorettes was maintained with an overall size of about 20 µm. Next, Gd(III) was added to the **NCoH** microflorettes with and without the DOTA-containing peptides. Again, SEM verified that the overall morphology of the microflorettes was not altered (Figure 3b–d), as compared to **NCoH** alone with Zn(II) (Figure 3a). From these SEM experiments, one may conclude that the addition of DOTA-containing peptides and Gd(III) does not disrupt the overall structure and surface morphology of the **NCoH** microflorettes.

To characterize the principal elements within the microflorettes, their chemical composition was analyzed by energy-dispersive X-ray spectroscopy (EDX) and inductively coupled plasma-mass spectrometry (ICP-MS). Microflorettes were generated as described above, with **NHdota** (5%) and **NCoH** (95%) with Zn(II) as the metal to trigger the self-assembly. After the addition of Gd(III), the microflorette particles were washed extensively to remove unbound metal ions. The particles were analyzed by EDX, and relative atomic abundances were obtained: carbon (80.46%), oxygen (19.06%), zinc (0.31%) and gadolinium (0.18%). These data verify the successful incorporation of both Zn(II) and Gd(III) during and after the formation of the microflorettes, respectively, at a ratio of 1.7:1.

ICP-MS was also used to quantify the content of zinc and gadolinium ions within microflorettes consisting of three different formulations of **NCoH**: pure **NCoH**, 5% **NHdota** and 5% **DOTA-His_6_**. Each peptide or peptide mix was initially exposed to Zn(II) to form florettes, followed by treatment with Gd(III) and extensive washing. Again, SEM confirmed the formation of microflorette structures of a similar size (~20 μm) and morphology (Figure 3b–d). Dissolution of the florettes was accomplished with aqua regia, and the samples were analyzed by ICP-MS. All formulations of the microflorettes were found to have comparable levels of zinc (Table 1). Gadolinium ions were not detected in a substantial amount for **NCoH** alone, due to the lack of a DOTA ligand as a coordination site (Table 1). However, the **NHdota** and **DOTA-His_6_** microflorettes contained notable amounts of gadolinium (Table 1). There is good agreement between the EDX and ICP-MS data for the ratio of Zn(II) to Gd(III) for the **NHdota**-containing microflorettes (1:1.7 and 1:1.6, respectively). Interestingly, the **DOTA-His_6_** florettes were found to contain about 40% more Gd(III) than those formed with **NHdota**. It is possible that the His-tag of **NHdota** can also contribute to the coordination of Gd(III), as imidazole-based ligands have been used for this metal ion [67,68], thereby increasing the loading of Gd(III) into the florettes.

**Figure 4:**
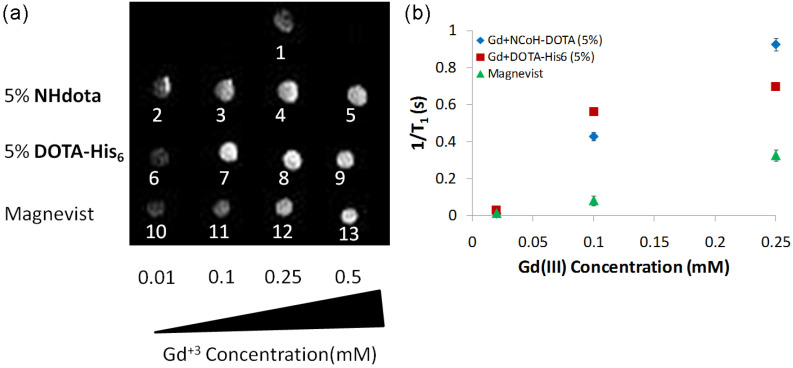
MRI analysis of microflorettes containing **NCoH** with **NHdota** (5%) and **DOTA-His_6_** (5%) with increasing concentration of Gd(III), compared to the commercially available Magnevist depicted as (**a**) T_1_-weighted images and (**b**) longitudinal relaxation rates (1/T_1_) versus the concentration of Gd(III). T_1_-wieghted images in (**a**) correspond to: 1—aragose in PBS containg 0.25 mM of Gd(III); 2–5—microflorettes containing 5% of **NHdota** and increasing concentrations of Gd(III); 6–9—microflorettes containing 5% of **DOTA-His_6_** and increasing concentrations of Gd(III); 10–13—commercially available Magnevist with increasing Gd(III) concentrations.

Since both the EDX and ICP-MS data verified the incorporation of Gd(III) ions within DOTA-containing microflorettes, the MRI analysis of the samples was next examined. We sought to determine if Gd(III)-loaded **NHdota** and **DOTA-His_6_** microflorettes increase the longitudinal relaxation (T_1_) of water protons, thereby enhancing the signal intensity in the MRI experiment. **NCoH**-based microflorettes composed of **NHdota** (5%) or **DOTA-His_6_** (5%) were formed as described above, followed by the addition of Gd(III). Since the particles are known to settle to the bottom of the tube upon standing in solution, they were suspended in agarose (1 wt%) in phosphate-buffered saline (PBS) (pH 7.4) so as to generate a homogenous distribution of the microflorettes throughout the sample volume. Agarose in PBS was also used as the negative control for this experiment. Each microflorette sample was examined on a 3T clinical MRI instrument to determine the MRI activity in a T_1_-weighted image and compared to the commercially available contrast agent Magnevist, which was also suspended in agarose in PBS (Figure 4), along with the longitudinal relaxivity (r_1_) being investigated.

By acquiring T_1_-weighted MRI images as a function of the amount of Gd(III) (0.01, 0.1, 0.25 mM) added to the **NCoH** florettes containing **NHdota** and **DOTA-His_6_**, in comparison to Magnevist, relaxivity values can be measured. With each florette sample, as the concentration of Gd(III) was increased, an increase in the MRI signal was observed up to 0.25 mM of Gd(III), whereas the Magnevist sample continued to increase up to 0.5 mM of Gd(III) (Figure 4a). For instance, a three-fold signal enhancement was observed for **DOTA-His_6_** particles, as compared to Magnevist, each with 0.1 mM of Gd(III) (Figure 4a, spots 7 and 11, respectively). These data provide evidence that **DOTA-His_6_** microflorettes are more efficient contrast agents compared to the clinically used Magnevist. From these data, relaxivity values (r_1_) were calculated from the slope of the inverse longitudinal relaxation time (1/T_1_) vs increasing Gd(III) concentrations (Figure 4b). The r_1_ values for **NHdota** and **DOTA-His_6_** microflorettes, along with Magnevist, were calculated to be 3.9 mM^−1^s^−1^, 2.7 mM^−1^s^−1^ and 1.3 mM^−1^s^−1^, respectively. The Gd(III)/DOTA-containing microflorettes produced a two- to three-fold higher relaxivity than Magnevist. This is potentially due to a slower rotational motion of these contrast agents, based on the considerable size increase in peptide-based microflorettes, as compared to discrete Gd(III) complexes. At the same time, since the size of the microflorettes containing **NHdota** and **DOTA-His_6_** is comparable, the differences in their relaxivity values may be due to different hydration states and water residency times for these materials. A larger relaxivity value is advantageous, since lower doses of the contrast agent could be used, thereby aiding medical use.

Since these systems are primarily intended for biological applications, it is critical that the Gd(III)-loaded microflorettes are biocompatible. Thus, in order to assess the biocompatibility of these microflorettes, an in vitro cytotoxicity assay was performed using HeLa cells. The cells were exposed to Gd(III)-loaded microflorettes containing up to 15% **NHdota** and 15% **DOTA-His_6_** for 24 h and compared to control cells without microflorettes. After 24 h, the viability of the cells was quantified via an MTS assay. The cells were found to be 92 and 98% viable, respectively, when treated with the two samples of microflorettes including **NHdota** and **DOTA-His_6_**. No appreciable cell toxicity was induced by Gd(III)-loaded microflorettes, making these particles suitable for in cyto experiments. Indeed, our previous study investigating the stability of the microflorettes in PBS and human plasma (55% in PBS) demonstrated the extensive stability of the particles in PBS (up to 2 months) and human plasma (1–3 days) [58]. Both the low cytotoxicity of the microflorettes and their stability in plasma bodes well for their use as imaging agents.

## 3. Materials and Methods

**General Peptide Synthesis.** A 10 mL peptide synthesis flask was loaded with 100 mg (0.55 mmol/g) of H-Rink Amide ChemMatrix resin. The resin was washed with CH_2_Cl_2_ and DMF. The desired Fmoc amino acid (6 eq, 0.33 mmol), O-benzotriazole-N,N,N’,N’-tetramethyl-uronium-hexafluoro-phosphate (HBTU) (6 eq, 0.33 mmol, 0.125 g), and diisopropylethylamine (DIEA) (12 eq, 0.66 mmol, 115 µL) in DMF (3 mL) were added to the reaction flask and rotated for 3 h at room temperature for coupling. Fmoc deprotection was achieved using 25% piperidine/DMF for 20 min. The solution was drained, and the resin was washed with DMF, CH_2_Cl_2_, methanol and CH_2_Cl_2_. This procedure was repeated until the full-length peptides were complete. The coupling of the NTA moiety [57] to the N-terminus (3 eq, 0.165 mmol, 71.1 mg) was achieved with HBTU (3 eq, 0.165 mmol, 62.7 mg) and DIEA (6 eq, 0.33 mmol, 57 µL) in DMF (2 mL) over 3 h at room temperature. For the **NHdota** peptide, the removal of the orthogonal Mtt-protecting group from the lysine side chain was performed with several washes of a 1% TFA solution in DCM. Coupling of the commercially available, protected and activated DOTA-mono-NHS-tris(t-Bu ester) ligand (from Macrocyclics) proceeded with DIEA in NMP for 4 h. Once the desired sequence was obtained, the resin was washed with MeOH and vacuum dried for 2 h. The final peptides were cleaved from the resin by treatment with a solution of 95% trifluoroacetic acid, 2.5% triisopropylsilane and 2.5% water for 3 h at room temperature. The cleavage solution was filtered through glass wool and the resin was washed with CH_2_Cl_2_. The solvent from the filtrate was removed in vacuo and the peptide was precipitated with cold diethyl ether (45 mL) at −20 °C for 4 h. The crude peptide was purified by reverse-phase HPLC using a semi-preparative Phenomenex C18 column with a linear 60 min solvent gradient, a flow rate of 10 mL/min and UV-Vis absorbance monitored at 214 nm and 254 nm. HPLC conditions for **NCoH**: gradient 2–25% (solvent A: CH_3_CN/0.1% TFA, solvent B: H_2_O/0.1% TFA), retention time 32.1 min; **NHdota**: gradient 8–30% (solvent A: CH_3_CN/0.1% TFA, solvent B: H_2_O/0.1% TFA), retention time 20.2 min; **DOTA-His_6_**: gradient 2–40% (solvent A: CH_3_OH/0.1% TFA, solvent B: H_2_O/0.1% TFA), retention time 23.7 min. Peptide purity was determined by analytical reverse-phase HPLC using an analytical Phenomenex C18 column with a linear 30 min gradient: **NCoH** 2–50%; **NHdota**: 5–30%; **DOTA-His_6_**; 2–30% with an eluent consisting of solvent A (CH_3_CN/0.05% TFA) and solvent B (H_2_O/0.05% TFA), a flow rate of 1.2 mL/min and UV-Vis absorbance monitored at 214 nm. All peptides were characterized by MALDI-TOF mass spectrometry (see Results and Discussion Section 2).

**Microflorette Formation:** For 5% **NHdota** microflorettes: solutions of **NCoH** (15 mM, 3.3 µL) and **NHdota** (1 mM, 2.5 µL) in MOPS buffer pH 7.1 (100 mM, 10 µL) were mixed with ZnCl_2_ (10 mM, 2 µL) and 32.2 µL of water, and the solutions were incubated for 24 h at room temperature. For 5% **DOTA-His_6_** microflorettes: solutions of **NCoH** (15 mM, 3.3 µL) and **DOTA-His_6_** (1 mM, 2.5 µL) in MOPS buffer pH 7.1 (100 mM, 10 µL) were mixed with ZnCl_2_ (10 mM, 2 µL) and 32.2 µL of water, and the solution was incubated for 24 h at room temperature. For **NCoH** microflorettes: a solution of **NCoH** (15 mM, 3.3 µL) in MOPS buffer pH 7.1 (100 mM, 10 µL) was mixed with ZnCl_2_ (10 mM, 2 µL) and 34.7 µL of water, and the solution was incubated for 24 h at room temperature. Following particle assembly, all solutions were centrifuged at 10,000× *g* for 5 min, the supernatant was carefully removed and the particles were resuspended in water (50 µL) and washed 3 times. For the introduction of Gd(III), after the last centrifugation, the supernatant was removed and MOPS pH 7.1 (20 mM, 47.5 µL) was added to the remaining microflorette pellet, followed by a solution of gadolinium chloride in water (10 mM, 2.5 µL); the mixture was incubated at room temperature for 24 h. The samples were washed 5 times in a similar manner as described above.

**Scanning Electron Microscopy:** Samples were imaged using an FEI Quanta 3D FEG SEM (FEI company, Hillsboro, OR, USA) using a ET detector and operating parameters of 5 kV, a spot size of 4, ~8–9 mm working distance and 10 K magnifications. Zinc- or gadolinium-loaded samples were prepared by air-drying the sample (2 µL) onto double-sided copper tape on a round glass cover slip. The cover slips were coated with Pt (2 × 60 s) prior to imaging.

**Energy-Dispersive X-ray Spectroscopy (EDX):** For 5% **NHdota** microflorettes, the Zn(II)- and Gd(III)-loaded samples were prepared in a similar procedure to that stated above. The final supernatant was resuspended in 30 µL of water, and 5 µL of the sample was placed on adhesive carbon tape and allowed to air dry for elemental analysis. The EDX elemental analysis was performed using the OXFORD INCA 250 electron-dispersive X-ray detector (EDX) operated with the FEI NOVA nanoSEM.

**Inductively Coupled Plasma-Mass Spectrometry (ICP-MS):** Following particle assembly with **NCoH**, 5% **NHdota** or 5% **DOTA-His_6_**, the number of spheres was counted on a hemocytometer, and ~5000 microflorettes were transferred to a 15 mL conical tube and digested in concentrated aqua regia solution (3:1 HCl: HNO_3_, 100 µL) overnight at 37 °C; then, they were placed on a mechanical shaker for 2 days at room temperature. Aqua regia (2%, in water, 4 mL) was added to the digested material, from which 1 mL aliquots were removed and analyzed by ICP-MS. A standard curve was generated by analyzing the increasing levels of zinc or gadolinium (purchased from Exaxol) using ICP-MS. The level of metal ions in the samples was determined using Thermo Scientific Element 2 ICP-MS based on the standard curves (in ppb), which were converted to μM for comparison.

**T_1_-weighted Image for Relaxivity Measurements:** Microflorettes were generated as described above. Before the gadolinium chloride was added, microflorette solutions of 5% **NHdota** and 5% **DOTA-His_6_** were divided into four equal parts and centrifuged. The supernatant was removed, and to the remaining microflorette pellets was added MOPS pH 7.1 buffer (20 mM, 49 µL, 45 µL, 37.5 µL, 25 µL), followed by an aqueous solution of GdCl_3_ (1 mM, 1 µL, 5 µL, 12.5 µL, 25 µL, respectively). The mixtures were incubated at room temperature for 24 h. The microflorette samples were washed with water, as described above, and resuspended in warm 1% agarose in PBS, vortexed and cooled to room temperature. Control solutions of diethylenetriaminepentaaceitc acid (10 mM, 5 µL) with GdCl_3_ (1 mM, 1 µL, 5 µL, 12.5 µL, 25 µL) (Magnevist) were prepared at room temperature for 24 h before being suspended in 1% agarose in PBS. The images were obtained on a 3T General Electric (Milwaukee, Brookfield, WI, USA) Signa HDx MR imager, with an 8-channel, high-definition knee array coil with Tr = 100, 200, 500, 1000, 2000, 4000 ms with TE = 10.04 ms. T_E_ = 20.08, 60.24, 100.24, 140.50, 180.72, 220.88, 261.04, 301.20 ms with T_R_ = 5000 ms held.

**MTS Cell Viability Assay:** The cellular toxicity of the microflorettes was examined using the 3-(4,5-dimethylthiazol-2-yl)-5-(3-carboxymethoxyphenyl)-2-(4-sulfophenyl)-2H-tetrazolium (MTS) cell viability assay. HeLa cells were seeded into 96-well plates at a density of 5000 cells/well in 200 µL of DMEM media supplemented with fetal bovine serum and grown in a humidified 5% CO_2_ atmosphere at 37 °C for 24 h. The cells were then incubated for 24 h in the presence of ~10,000 microflorettes per well. Following incubation, 20 µL of CellTiter 96 Aqueous One solution was added to each well, and the cells were incubated for an additional 2.5 h. The absorbance of each well was read with a TECAN SPECTRAFluor Plus fluorescence plate reader at 492 nm. For each experiment, a control of cells that were not incubated with microflorettes was also analyzed. The average absorbance for each sample was calculated, and the percent viability was determined using the following equation: % cell viability = *A*_492_ treated cells/*A*_492_ untreated cells × 100.

## 4. Conclusions

Here, we developed a facile means to incorporate DOTA-based ligands within spherical assemblies (microflorettes) formed from the collagen mimetic peptides **NCoH** and Zn(II). This was achieved by introducing DOTA-containing peptides during the **NCoH** assembly process using two different approaches: in one case, the DOTA was introduced into a sidechain of **NCoH** (**NHdota**); in the other case, a His-tagged DOTA peptide (**DOTA-His_6_**) was used. In each of these scenarios, we observed that introduction of the DOTA-containing peptide (5%) did not inhibit **NCoH** microflorette formation. Interestingly, microflorettes did not form when Gd(III) was used in place of Zn(II), and Gd(III) uptake was minimal in pre-formed microflorettes. However, Gd(III) could be introduced within the microflorettes formed when the DOTA-containing peptides were present within the assemblies. We observed that the **DOTA-His_6_** method allowed for a measurable increase in the uptake of Gd(III) within the florettes, as compared to having the DOTA group attached to the peptide sidechain in **NHdota**. The MRI studies of DOTA-containing microflorettes demonstrated that the peptide-based assemblies, doped with Gd(III) ions, possess higher longitudinal relaxivity values (r_1_) in comparison to clinically used Gd(III) complexes, opening up their use as candidates for bioimaging. The increase in relaxivity observed with the DOTA-containing microflorettes is likely associated with the considerable decrease in the rotational motion of these assemblies. Previous reports have demonstrated that the core and surface of microflorettes can be decorated with His-tagged proteins [58]. Therefore, it may be feasible to introduce cell- and tissue-targeting proteins and peptides on the surface of the microflorettes to add extra benefit to the use of these assemblies in MRI.

## Figures and Tables

**Table 1:** Analysis of zinc and gadolinium ions within microflorettes by ICP-MS.

Peptide	[Zn] (µM) ^a^	[Gd] (µM) ^a^	Ratio [Zn]:[Gd]
**NCoH**	0.165	0.003	55:1
**5% NHdota**	0.173	0.111	1.6:1
**5% DOTA-His_6_**	0.156	0.158	1:1

^a^ Based on dissolution of ~5000 microflorettes for each sample.

## Data Availability

Data is available from the corresponding author.

## Sample Availability

Samples of the compounds described herein are available from the authors.
